# 

**Published:** 1857-01-01

**Authors:** 


					THE JOURNAL
? ) ><
OF
PSYCHOLOGICAL MEDICINE
MENTAL PATHOLOGY.
EDITED BY
FORBES WIN SLOW, M. D., D. C. L. Oxon
VOL. X.
C/. ; ? ?,
9 o*
LONDON:
JOHN CHURCHILL, NEW BURLINGTON STREET.
MDCCCLVII.
0 3
O rT
f\ f!
LONDON:
SAVIll AND BDWABDS, PEINTEES, CHANDOS STEBBT,
COVBNT GAEDBN.

				

